# Real world treatment patterns for recurrent and metastatic head and neck cancer in the post-KEYNOTE 048 era

**DOI:** 10.3389/fonc.2025.1577509

**Published:** 2025-05-02

**Authors:** Daniel Y. Lee, Alexander Pan, Maxim Yaskolko, Michael Chiorazzi, Aarti Bhatia, Barbara Burtness, Jeffrey J. Ishizuka

**Affiliations:** ^1^ Department of Internal Medicine (Oncology), Smilow Cancer Center at Yale New Haven Hospital, New Haven, CT, United States; ^2^ Department of Pathology, Yale School of Medicine, New Haven, CT, United States; ^3^ Department of Immunobiology, Yale School of Medicine, New Haven, CT, United States

**Keywords:** KEYNOTE-048, head and neck cancer, immunotherapy, pembrolizumab, cetuximab

## Abstract

**Background:**

While KEYNOTE-048 established anti-PD1 with or without chemotherapy as first-line treatment for recurrent and metastatic head and neck squamous cell carcinoma (HNSCC) with combined positive score (CPS) ≥ 1, treatment choice remains ambiguous given additional toxicity of combination treatment.

**Methods:**

Patients treated first-line with anti-PD1 monotherapy, anti-PD1+chemotherapy, or cetuximab+chemotherapy in the Flatiron Health database were included. Treatment group differences were assessed with chi-squared and t-tests, and selection factors were analyzed with logistic regressions. Survival was assessed with Kaplan-Meier curves, log-rank tests, and Cox regressions.

**Results:**

Of 2577 patients included, Anti-PD1 monotherapy (n=1410) improved survival over cetuximab+chemotherapy (n=577, median survival 14.6 vs. 12.6 months, p=0.015), while anti-PD1+chemotherapy (n=590) showed a nonsignificant trend towards improvement (median survival 14.3 vs. 12.6 months, p=0.053). In HPV-associated disease, survival was equal between regimens. Addition of chemotherapy improved survival over anti-PD1 monotherapy in non-HPV associated tumors with CPS 1-9 (median survival 18.0 vs. 10.3 months, p=0.029) and in oral cavity primaries (median survival 10.3 vs. 7.6 months, p=0.003).

**Conclusions:**

Subgroups of patients with recurrent or metastatic HNSCC, including non-HPV associated disease with CPS 1-9 and oral cavity primaries, may derive benefits from the addition of chemotherapy to anti-PD1 therapy.

## Introduction

The randomized phase III trial KEYNOTE-048 (KN048) established anti-PD1 plus platinum-5-fluorouracil chemotherapy (combination therapy) or anti-PD1 monotherapy as the front-line treatment for patients with recurrent or metastatic head and neck squamous cell carcinoma (HNSCC) and tumor combined positive score (CPS) ≥1 over cetuximab plus platinum-based chemotherapy (1, 2). Alternative chemotherapy backbones substitute a taxane for 5-fluorouracil (3). However, KN048 was not powered to directly compare anti-PD1 plus chemotherapy versus anti-PD1 monotherapy, and the choice between the two regimens remains ambiguous for some patients with CPS ≥1.

The choice between pembrolizumab monotherapy and pembrolizumab-chemotherapy combination is currently guided by CPS, performance status, clinical symptom burden, and patient preference. Combination therapy led to a higher rate of adverse events in KN048, with grade ≥ 3 adverse events occurring in 85% of patients in the combination therapy group and in 55% of patients in the pembrolizumab monotherapy group (1). However for patients with tumor CPS 1-19, subset analyses of KN048 suggested improved survival with combination therapy but not anti-PD1 monotherapy, albeit this study was not powered for this prospective analysis (4). This potential benefit of combination therapy over anti-PD1 monotherapy is also suggested by long term follow up data of KN048 and by studies showing a pan-cancer synergy between platinum chemotherapy and immune checkpoint inhibitors (2, 5). Furthermore, patients with highly symptomatic or rapidly progressive disease may benefit from combination therapy given the higher objective response rate, lower rate of early disease progression, and longer progression-free survival compared to anti-PD1 monotherapy (1, 2). It remains unclear what factors other than CPS - such as HPV status, primary tumor site or tumor mutation burden (TMB) - should impact the choice between these two approaches.

In this study, we utilize a national US oncology dataset to assess real world practice patterns and outcomes in the treatment of recurrent or metastatic HNSCC. We sought to examine the activity of anti-PD1 therapy in HNSCC in a non-clinical trial setting and evaluate which subsets of patients most benefit from the addition of chemotherapy to anti-PD1 therapy. Additionally, we describe factors associated with the receipt of combination therapy over anti-PD1 monotherapy in a real-world data set.

## Methods

### Patient cohort

We performed a retrospective cohort study utilizing the Flatiron Health database, an electronic health record (EHR) based de-identified dataset that includes data from approximately 280 cancer clinics (~800 sites of care) in the United States. Yale University institutional review board approval was obtained prior to conducting the study (IRB#2000036076). Patients age >18 treated with cetuximab plus platinum-based chemotherapy, anti-PD1 (pembrolizumab or nivolumab) monotherapy, or anti-PD1 plus platinum-based chemotherapy in the front line setting for locally recurrent or metastatic HNSCC between 1/1/2011 and 9/30/2023 were included in this study. Patients with recurrent disease were not candidates for curative intent radiation or surgical management. Patients were excluded if they lacked survival data, lacked follow up time after front line systemic treatment initiation, or received definitive radiation within 6 months of systemic front-line treatment for locally recurrent or metastatic disease (used as a surrogate for concurrent chemotherapy as a part of radiation). Chemotherapy regimens included platinum plus 5-fluorouracil based regimens and platinum plus taxane regimens (**Supplementary Tables 1, 2**).

### Data

Patient specific variables included baseline demographics (age, sex, race, ethnicity, socioeconomic status [SES], smoking status) and Eastern Cooperative Oncology Group [ECOG] performance status at first-line treatment initiation. Disease-specific variables included site of primary tumor (oropharynx, oral cavity, larynx, hypopharynx, or head and neck cancer with unknown primary), human papilloma virus [HPV] status, criteria for advanced disease (locoregionally recurrent versus distant metastatic disease), and combined positive score [CPS] (subgroups of <1, 1-9, 10-19, and ≥20). Tumors were considered HPV-associated if the primary site was oropharynx and HPV test was positive; non-oropharyngeal primaries were presumed as non-HPV associated. Treatment-specific variables included site of clinical care (academic versus community) and treatment regimen. The primary outcome was overall survival which was defined as the time from first-line treatment initiation in the advanced setting to death, censoring at last follow up or data cutoff (September 30, 2023). Death was defined from a composite mortality variable developed by Flatiron Health using structured and unstructured EHR-derived data linked to a commercial death data source and US Social Security Death Index (6).

### Statistical analysis

Baseline patient, treatment, and disease variables were characterized using descriptive statistics and compared by treatment group utilizing Pearson chi-squared test for categorical variables and unpaired t-test for continuous variables. Univariable and multivariable logistic regression models were utilized to assess factors associated with receipt of anti-PD1 plus chemotherapy over anti-PD1 monotherapy. Kaplan-Meier curves were used to estimate overall survival, and survival between treatment groups were compared by log-rank test for the overall population and subgroups including by HPV status, CPS status, and primary tumor site. Univariable and multivariable cox regression models were utilized to estimate adjusted hazard ratios (HRs) for overall survival in the total cohort. For all multivariable models, models were adjusted utilizing variables with p < 0.10 on univariable analysis. Statistical significance was defined as a 2-sided p value < 0.05. Statistical analyses were performed using Stata SE, version 15.0 (StataCorp, College Station, TX) and Graphpad Prism 9 (GraphPad Software, Boston, Massachusetts USA).

## Results

### Patient cohort

Overall, 2577 patients met inclusion criteria (**Supplementary Figure 1**). In the overall cohort, the median age was 66 (range 20 – 85), and most patients were male (79.5%) and white (67.5%, **Table 1**). Most patients were treated at non-academic institutions (78.9%). In this cohort, 60.4% of patients had non-HPV associated disease, 32.6% had HPV-associated disease, and 7.0% had unknown HPV status or primary tumor site. 1410 patients (54.7%) were treated with anti-PD1 monotherapy, 577 (22.4%) with combination anti-PD1 plus chemotherapy, and 590 (22.9%) with cetuximab plus chemotherapy in the front-line setting for recurrent or metastatic disease. Of the overall cohort, 43.8% of patients were recorded to have received subsequent lines of anti-cancer therapy.

**Table 1:** Patient demographics and disease characteristics.

	Anti-PD1 alone	Anti-PD1 + Chemo	Cetux + Chemo	Chi2 p-value
**Patients**	1,410	577	590	
**Median Age (range)**	68 (20 - 85)	64 (29 - 85)	62 (31 – 85)	
Sex				0.055
** Male**	1,096 (77.7%)	471 (81.6%)	481 (81.5%)	
** Female**	314 (22.3%)	106 (18.4%)	109 (18.5%)	
Race				< 0.001
** White**	926 (65.7%)	400 (69.3%)	413 (70.0%)	
** Black**	78 (5.5%)	23 (4.0%)	43 (7.3%)	
** Asian**	16 (1.1%)	9 (1.6%)	7 (1.2%)	
** Other**	259 (18.4%)	64 (11.1%)	86 (14.6%)	
** Unknown**	131 (9.3%)	81 (14.0%)	41 (6.9%)	
Ethnicity				< 0.001
** Non-Hispanic**	1,048 (74.3%)	423 (73.3%)	483 (81.9%)	
** Hispanic**	64 (4.6%)	33 (5.7%)	37 (6.3%)	
** Unknown**	298 (21.1%)	121 (21.0%)	70 (11.8%)	
Practice Type				0.269
** Community**	1,129 (80.1%)	448 (77.6%)	456 (77.3%)	
** Academic**	281 (19.9%)	129 (22.4%)	134 (22.7%)	
Socioeconomic Status				0.422
** Lowest Quintile**	235 (16.7%)	81 (14.0%)	89 (15.1%)	
** 2nd Quintile**	295 (20.9%)	125 (21.7%)	119 (20.2%)	
** 3rd Quintile**	298 (21.1%)	120 (20.8%)	116 (19.6%)	
** 4th Quintile**	269 (19.1%)	129 (22.4%)	127 (21.5%)	
** Highest Quintile**	191 (13.6%)	87 (15.1%)	86 (14.6%)	
** Unknown**	122 (8.6%)	35 (6.0%)	53 (9.0%)	
Baseline ECOG				< 0.001
** 0**	364 (25.8%)	179 (31.0%)	162 (27.4%)	
** 1**	613 (43.5%)	272 (47.2%)	230 (39.0%)	
** 2+**	240 (17.0%)	71 (12.3%)	86 (14.6%)	
** Unknown**	193 (13.7%)	55 (9.5%)	112 (19.0%)	
Site of Primary				0.268
** Oropharynx**	721 (51.1%)	283 (49.1%)	307 (52.0%)	
** Oral Cavity**	272 (19.3%)	125 (21.7%)	110 (18.6%)	
** Larynx**	297 (21.1%)	126 (21.8%)	112 (19.0%)	
** Hypopharynx**	80 (5.7%)	36 (6.2%)	40 (6.8%)	
** Tongue/Pharynx NOS**	11 (0.8%)	0 (0%)	6 (1.0%)	
** H&N Unknown Primary**	29 (2.0%)	7 (1.2%)	15 (2.6%)	
Group Stage at Diagnosis				< 0.001
** Stage I**	93 (6.6%)	43 (7.4%)	27 (4.6%)	
** Stage II**	158 (11.2%)	60 (10.4%)	33 (5.6%)	
** Stage III**	223 (15.8%)	88 (15.2%)	59 (10.0%)	
** Stage IV (unspecified)**	31 (2.2%)	39 (6.8%)	10 (1.7%)	
** Stage IVA**	555 (39.4%)	144 (25.0%)	208 (35.3%)	
** Stage IVB**	77 (5.5%)	28 (4.8%)	35 (5.9%)	
** Stage IVC**	79 (5.6%)	106 (18.4%)	150 (25.4%)	
** Unknown**	194 (13.7%)	69 (12.0%)	68 (11.5%)	
Advanced Criteria				< 0.001
** Distant Metastatic**	789 (56.0%)	375 (65.0%)	365 (61.9%)	
** Locoregional Recurrence**	621 (44.0%)	202 (35.0%)	225 (38.1%)	
HPV Association				< 0.001
** HPV associated**	485 (34.4%)	185 (32.1%)	169 (28.6%)	
** Non-HPV associated**	842 (59.7%)	369 (64.0%)	346 (58.6%)	
** Unknown**	83 (5.9%)	23 (4.0%)	75 (12.7%)	
Smoking Status				0.017
** History of Smoking**	1,090 (77.3%)	433 (75.0%)	474 (80.4%)	
** No History of Smoking**	320 (22.7%)	144 (25.0%)	114 (19.3%)	
** Unknown**	0 (0.0%)	0 (0.0%)	2 (0.3%)	
Primary Surgery				0.415
** Yes**	459 (32.6%)	172 (29.8%)	180 (30.5%)	
** No**	951 (67.4%)	405 (70.2%)	410 (69.5%)	
Primary Radiation				< 0.001
** Yes**	1,110 (78.7%)	355 (61.5%)	347 (58.1%)	
** No**	300 (21.3%)	222 (38.5%)	243 (41.2%)	
CPS				< 0.001
** < 1**	303 (21.5%)	181 (31.4%)	67 (11.4%)	
** 1-9**	133 (9.4%)	99 (17.1%)	10 (1.7%)	
** 10-19**	79 (5.6%)	38 (6.6%)	1 (0.2%)	
** (20**	228 (16.2%)	91 (15.8%)	8 (1.3%)	
** Unknown**	667 (47.3%)	168 (29.1%)	504 (85.4%)	

### Real world treatment patterns

**Figure 1:**
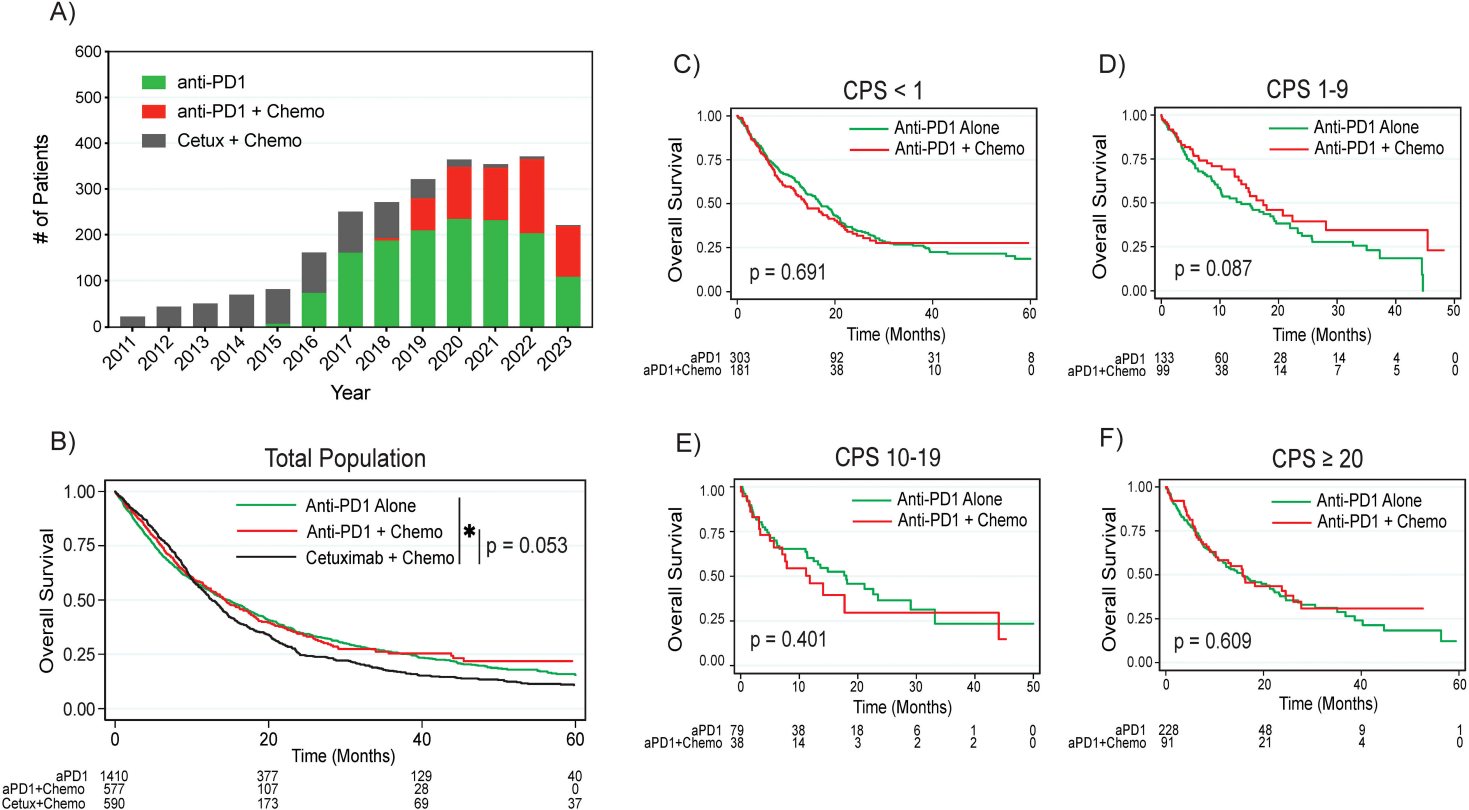
Treatment patterns and overall survival by treatment regimen. **(A)** Treatment regimen frequency by year. **(B)** Overall survival by treatment regimen by combined positive score in the **(C)** CPS <1, **(D)** CPS 1-9, **(E)** CPS 10-19, and **(F)** CPS ≥20 populations. P values are based on log rank test. Asterisk denotes p < 0.05. HPV, human papilloma virus; CPS, combined positive score; aPD1, anti-PD1; Cetux, Cetuximab; Chemo, chemotherapy.

More patients in this cohort were treated with anti-PD1 monotherapy compared to combined anti-PD1 plus chemotherapy, although an increasing proportion of patients were treated with combination therapy post-2019 (**Figure 1A**). This predominance of anti-PD1 monotherapy applied to both the CPS <1 subgroup, where 62.6% of patients were treated with anti-PD1 monotherapy as opposed to combination anti-PD1 plus chemotherapy, and in the CPS 1 to 19 group, where 60.7% were treated with anti-PD1 monotherapy. Of the 303 patients with CPS <1 treated with anti-PD1 monotherapy, 80.9% of these patients were documented to have received prior definitive radiation.

Out of 2577 patients, 1128 patients (43.8%) were reported to have received second line treatment (**Supplementary Tables 3, 4**). Of the 365 patients who received second line treatment after front line treatment with Cetuximab + chemotherapy, 191 (52.3%) of patients received an immunotherapy containing regimen.

On multivariable logistic regression, notable factors associated with decreased likelihood of receiving combination therapy compared to anti-PD1 monotherapy included older age (odds ratio [OR] 0.97 per year, 95% confidence interval [CI] 0.96 – 0.98, p < 0.001), female gender (OR 0.76, 95% CI 0.58 – 0.99, p = 0.042), other race (OR 0.60, 95% CI 0.44 – 0.82, p = 0.001), ECOG performance status of 2 or more compared to 0 (OR 0.68, 95% CI 0.48 – 0.95, p = 0.025), locoregional recurrence as opposed to distant metastatic disease (OR 0.77, 95% CI 0.62 – 0.96, p = 0.020), and CPS ≥20 (OR 0.66, 95% CI 0.47 – 0.91, p = 0.011, **Table 2**). Factors associated with increased likelihood of receiving combination anti-PD1 plus chemotherapy included higher SES (OR 1.52 for 4^th^ quintile compared to lowest, 95% CI 1.07 – 2.16, p = 0.019) and no history of prior definitive radiation (OR 2.46, 95% CI 1.96 – 3.10, p < 0.001). Primary tumor site, tumor HPV association, and practice type did not impact the treatment regimen patients received.

**Table 2:** Predictors of Chemo + anti-PD1 versus anti-PD1 alone.

	Univariate		Multivariate	
	OR (95% CI)	p value	OR (95% CI)	p value
**Age (per year)**	0.97 (0.96 – 0.98)	**< 0.001**	0.97 (0.96 – 0.98)	**< 0.001**
Sex				
** Male**	Reference		Reference	
** Female**	0.79 (0.61 – 1.00)	**0.054**	0.76 (0.58 – 0.99)	**0.042**
Race				
** White**	Reference		Reference	
** Black**	0.68 (0.42 – 1.10)	0.119	0.74 (0.44 – 1.24)	0.249
** Asian**	1.30 (0.57 – 2.97)	0.530	1.47 (0.62 – 3.52)	0.385
** Other**	0.57 (0.42 – 0.77)	**< 0.001**	0.60 (0.44 – 0.82)	**0.001**
**vUnknown**	1.43 (1.06 – 1.93)	**0.019**	1.54 (1.12 – 2.13)	**0.008**
Ethnicity				
** Non-Hispanic**	Reference			
** Hispanic**	1.28 (0.83 – 1.97)	0.270		
** Unknown**	1.01 (0.79 – 1.28)	0.961		
Practice Type				
** Community**	Reference			
** Academic**	1.16 (0.91 – 1.46)	0.225		
Socioeconomic Status				
** Lowest Quintile**	Reference		Reference	
** 2^nd^ Quintile**	1.23 (0.86 – 1.71)	0.217	1.33 (0.94 – 1.89)	0.110
** 3^rd^ Quintile**	1.17 (0.84 – 1.62)	0.355	1.23 (0.86 – 1.75)	0.256
** 4^th^ Quintile**	1.39 (1.00 – 1.93)	**0.049**	1.52 (1.07 – 2.16)	**0.019**
** Highest Quintile**	1.32 (0.92 – 1.89)	0.127	1.37 (0.93 – 2.02)	0.110
** Unknown**	0.83 (0.53 – 1.31)	0.427	0.90 (0.56 – 1.45)	0.662
Baseline ECOG				
** 0**	Reference		Reference	
** 1**	0.90 (0.72 – 1.13)	0.379	0.98 (0.77 – 1.26)	0.907
** 2+**	0.60 (0.44 – 0.83)	**0.002**	0.68 (0.48 – 0.95)	**0.025**
** Unknown**	0.58 (0.41 – 0.82)	**0.002**	0.61 (0.42 – 0.88)	**0.008**
Site of Primary				
** Oropharynx**	Reference			
** Oral Cavity**	1.17 (0.91 – 1.51)	0.221		
** Larynx**	1.08 (0.84 – 1.39)	0.542		
** Hypopharynx**	1.15 (0.76 – 1.74)	0.520		
** H&N Unknown Primary**	0.61 (0.27 – 1.42)	0.255		
Advanced Criteria				
** Distant Metastatic**	Reference		Reference	
** Locoregional Recurrence**	0.68 (0.56 – 0.84)	**< 0.001**	0.77 (0.62 – 0.96)	**0.020**
HPV Association				
** HPV associated**	Reference			
** Non-HPV associated**	1.15 (0.93 – 1.42)	0.193		
** Unknown**	0.73 (0.44 – 1.19)	0.203		
Smoking Status				
** Smoker**	Reference			
** Non-Smoker**	1.13 (0.90 – 1.42)	0.280		
Primary Surgery				
** Yes**	Reference			
** No**	1.14 (0.92 – 1.40)	0.233		
Primary Radiation				
** Yes**	Reference		Reference	
** No**	2.31 (1.87 – 2.86)	**< 0.001**	2.46 (1.96 – 3.10)	**< 0.001**
CPS				
** < 1**	Reference		Reference	
** 1-9**	1.25 (0.91 – 1.71)	0.176	1.18 (0.84 – 1.65)	0.347
** 10-19**	0.81 (0.52 – 1.24)	0.322	0.71 (0.45 – 1.12)	0.140
** ≥20**	0.67 (0.49 – 0.91)	**0.010**	0.66 (0.47 – 0.91)	**0.011**
** NA**	0.60 (0.50 – 0.72)	**< 0.001**	0.43 (0.33 – 0.56)	**< 0.001**

Bolded values represent statistically significant p-values at a threshold of p < 0.05.

### Overall survival stratified by HPV status and CPS

The median follow-up interval from initiation of front-line treatment was 9.7 months (range 0.03 – 131 months). In the overall cohort, patients treated with anti-PD1 monotherapy had significantly improved survival compared to patients treated with cetuximab plus chemotherapy (median survival 14.6 months versus 12.6 months, p = 0.015 on log rank test, **Figure 1B**). Patients treated with anti-PD1 plus chemotherapy trended towards improved survival compared to cetuximab plus chemotherapy (median survival 14.3 months versus 12.6 months, p = 0.053). When comparing survival between anti-PD1 monotherapy and combination anti-PD1 plus chemotherapy, there were no significant differences in survival between the two treatment approaches either in the overall cohort (p = 0.611) or by CPS subgroups, although there was a trend towards improved survival with combination treatment in the CPS 1-9 group (median survival 17.4 months with combination versus 13.6 months with monotherapy, p = 0.087, **Figures 1C–F**, **Supplementary Figure 2**).

**Figure 2:**
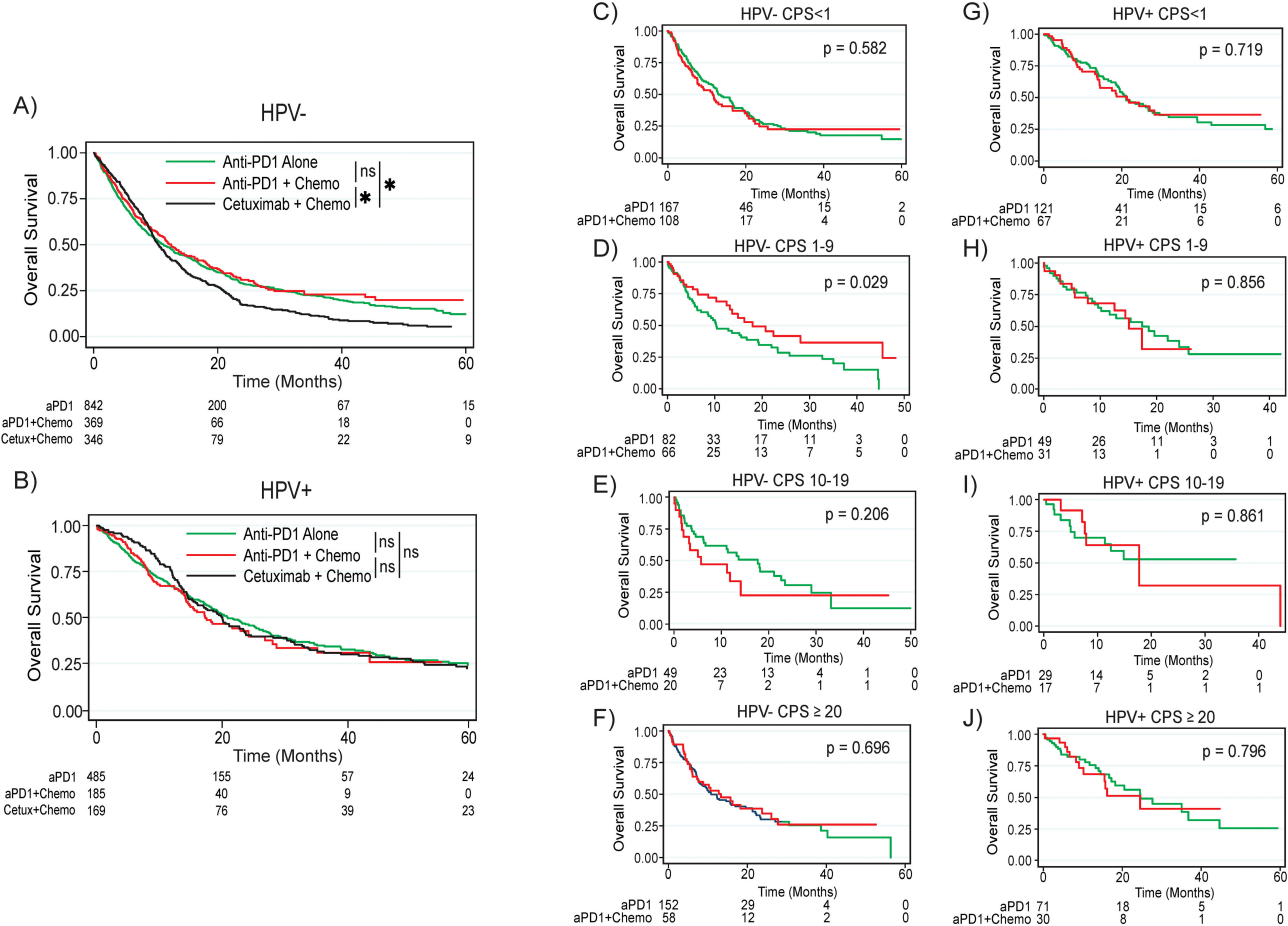
Overall survival by treatment regimen based on HPV status and CPS. **(A)** Overall survival by treatment regimen for the overall non-HPV associated population. **(B)** Overall survival by treatment regimen for the overall HPV associated population. For the non-HPV associated population, overall survival for the **(C)** CPS <1, **(D)** CPS 1-9, **(E)** CPS 10-19, and **(F)** CPS ≥20 populations. For the HPV associated population, overall survival for the **(G)** CPS <1, **(H)** CPS 1-19, **(I)** CPS 10-19, and **(J)** CPS ≥20 populations. P values are based on log rank test. Asterisk denotes p < 0.05. ns, not significant; HPV, human papilloma virus; CPS, combined positive score; aPD1, anti-PD1; Cetux, Cetuximab; Chemo, chemotherapy.

When comparing treatment outcomes in patients with non-HPV associated tumors, patients treated with either anti-PD1 monotherapy (median survival 11.2 months, p = 0.011) or combination anti-PD1 plus chemotherapy (median survival 12.5 months, p = 0.006) had significantly improved survival compared to patients treated with cetuximab plus chemotherapy (median survival 10.4 months); there was no significant difference in survival between anti-PD1 monotherapy and combination anti-PD1 plus chemotherapy (p = 0.290, **Figure 2A**). Interestingly, patients with non-HPV associated tumors and CPS 1-9 had significantly improved survival after treatment with combination anti-PD1 plus chemotherapy compared to anti-PD1 monotherapy (median survival 18.0 months versus 10.3 months, p = 0.029, **Figure 2D**, **Supplementary Figure 2**). When comparing survival by CPS (<1, 1-9, and ≥10) within each treatment regimen (anti-PD1 monotherapy or anti-PD1 plus chemotherapy) in the overall population as well as patients with HPV associated and non-HPV associated tumors, there were no significant differences in survival by CPS (**Supplementary Figure 3**).

In contrast, patients with HPV associated tumors did not have significantly improved survival after treatment with either anti-PD1 monotherapy (median survival 21.1 months, p = 0.972) or combination anti-PD1 plus chemotherapy (median survival 17.5 months, p = 0.381) compared to patients treated with cetuximab plus chemotherapy (median survival 20.2 months, **Figure 2B**). There was no difference in survival between anti-PD1 monotherapy and combination anti-PD1 plus chemotherapy among all patients with HPV associated tumors (p = 0.556). When looking at survival by CPS, there were no survival differences between patients treated with anti-PD1 monotherapy versus combination anti-PD1 plus chemotherapy for any CPS subgroup (**Figures 2D-F, G-J**).

### Survival by primary tumor site

**Figure 3:**
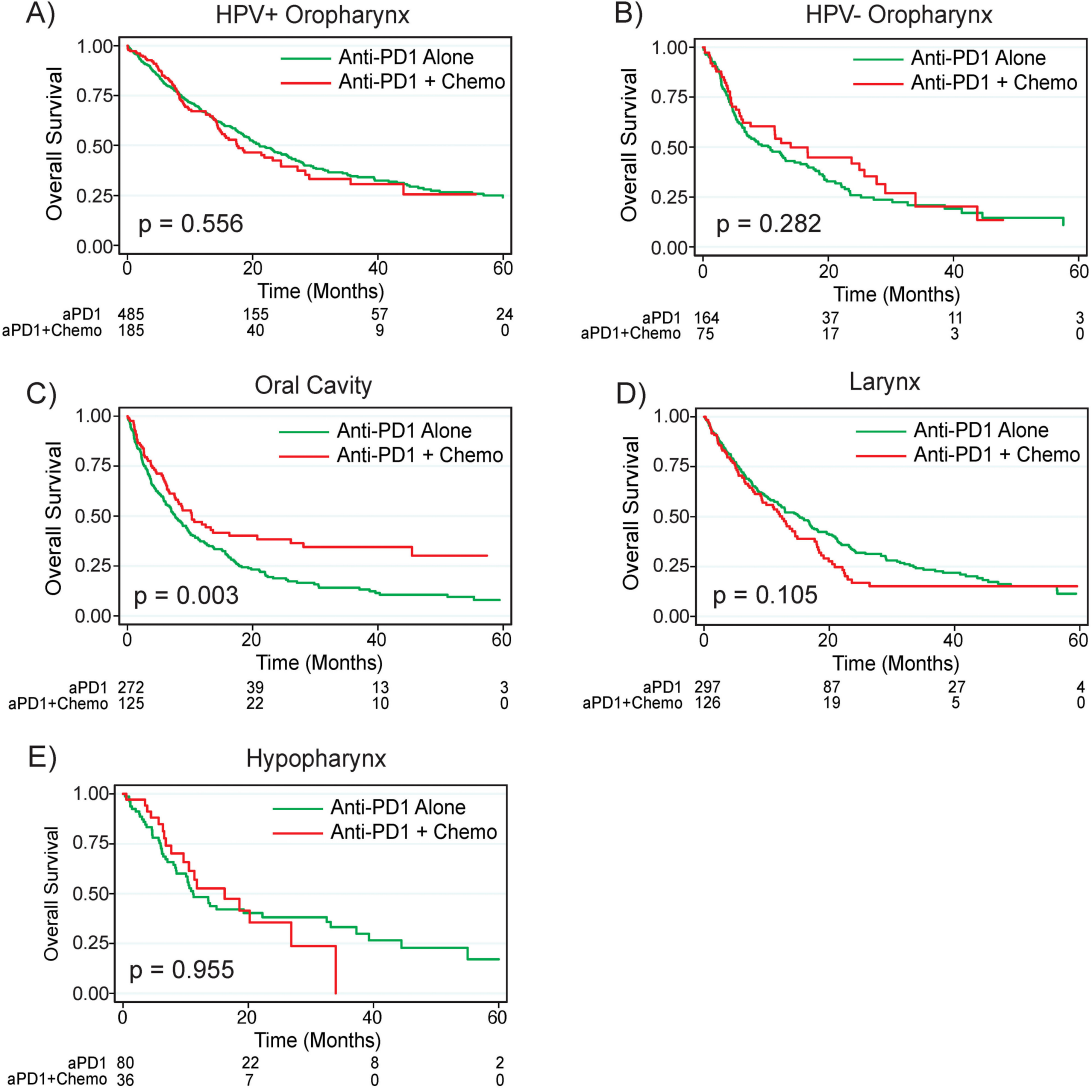
Anti-PD1 plus chemotherapy improves survival for patients with Oral Cavity primaries. Overall survival by treatment regimen based on primary tumor site for tumors of the **(A)** HPV+ oropharynx, **(B)** HPV- oropharynx, **(C)** Oral Cavity, **(D)** Larynx, and **(E)** Hypopharynx. P values are based on log rank test. HPV, human papilloma virus; CPS, combined positive score; aPD1, anti-PD1; Chemo, chemotherapy.

Most patients in the overall cohort had primary tumors of the oropharynx (50.9%) followed by tumors of the larynx (20.8%), oral cavity (19.7%), hypopharynx (6.1%), and head and neck cancer with unknown primary (2.0%). Of patients treated with anti-PD1 monotherapy or combination anti-PD1 plus chemotherapy, patients with oral cavity primary tumors (median survival 8.5 months) had worse survival compared to patients with laryngeal (median survival 13.7 months, p = 0.002), hypopharyngeal (median survival 11.8, p = 0.005), and head and neck cancer with unknown primaries (median survival 36.0 months, p < 0.001); there was a trend towards worse survival for patients with oral cavity primary tumors compared to non-HPV associated oropharyngeal primaries (median survival 8.5 months vs 11.5 months, p = 0.069, **Supplementary Figure 4**). Interestingly, patients with primary tumors of the oral cavity treated with combination anti-PD1 plus chemotherapy had significantly improved survival compared to those treated with anti-PD1 monotherapy (median survival 10.3 months versus 7.6 months, p = 0.003) while there were no significant differences in survival between the two treatment regimens for patients with primary tumors of the oropharynx, larynx, or hypopharynx (**Figure 3**).

### Multivariable survival model

On multivariable cox regression for patients treated with immunotherapy, notable factors associated with worse overall survival included older age (HR 1.01 per year, 95% CI 1.00 – 1.01, p = 0.046), ECOG score of 1 (HR 1.34, 95% CI 1.15 – 1.55, p < 0.001) or 2+ (HR 2.17, 95% CI 1.82 – 2.60, p < 0.001) compared to 0, and non-HPV associated disease (HR 1.48, 95% CI 1.21 – 1.80, p < 0.001, **Table 3**). On multivariable analysis, primary tumors of the oral cavity were associated with worse survival compared to primary tumors of the larynx (HR 1.36, 95% CI 1.12 – 1.66, p = 0.002) and hypopharynx (HR 1.36, 95% CI 1.02 – 1.81, p = 0.035). Variables associated with improved survival on multivariable analysis included Hispanic ethnicity (HR 0.57, 95% 0.42 – 0.78, p < 0.001), higher socioeconomic status (HR 0.66 for the highest quintile compared to the lowest, 95% CI 0.53 – 0.83, p < 0.001), and head and neck cancer with unknown primary (HR 0.40 compared to oropharyngeal primary, 95% CI 0.24 – 0.69, p = 0.001).

**Table 3:** Predictors of survival for ICI treated patients (anti-PD1 +/- chemo).

	Univariate		Multivariate	
	HR (95% CI)	p value	HR (95% CI)	p value
**Age (per year)**	1.01 (1.00 – 1.02)	**0.002**	1.01 (1.00 – 1.01)	**0.046**
Sex				
**Male**	Reference			
**Female**	1.09 (0.95 – 1.25)	0.197		
Race				
**White**	Reference			
**Black**	1.06 (0.83 – 1.36)	0.635		
**Asian**	1.08 (0.65 – 1.81)	0.757		
**Other**	1.07 (0.92 – 1.25)	0.391		
**Unknown**	0.95 (0.77 – 1.16)	0.588		
Ethnicity				
**Non-Hispanic**	Reference		Reference	
**Hispanic**	0.66 (0.49 – 0.89)	**0.006**	0.57 (0.42 – 0.78)	**< 0.001**
**Unknown**	0.94 (0.82 – 1.09)	0.418	0.94 (0.81 – 1.09)	0.390
Practice Type				
**Community**	Reference			
**Academic**	0.94 (0.82 – 1.09)	0.422		
Socioeconomic Status				
**Lowest Quintile**	Reference		Reference	
**2^nd^ Quintile**	1.10 (0.91 – 1.32)	0.327	1.02 (0.85 – 1.23)	0.824
**3^rd^ Quintile**	0.89 (0.74 – 1.08)	0.229	0.87 (0.71 – 1.05)	0.141
**4^th^ Quintile**	0.88 (0.73 – 1.07)	0.199	0.89 (0.73 – 1.08)	0.228
**Highest Quintile**	0.74 (0.59 – 0.91)	**0.005**	0.66 (0.53 – 0.83)	**< 0.001**
**Unknown**	1.12 (0.89 – 1.42)	0.343	1.07 (0.84 – 1.36)	0.574
Baseline ECOG				
**0**	Reference		Reference	
**1**	1.43 (1.24 – 1.66)	**< 0.001**	1.34 (1.15 – 1.55)	**< 0.001**
**2+**	2.50 (2.10 – 2.96)	**< 0.001**	2.17 (1.82 – 2.60)	**< 0.001**
**Unknown**	1.28 (1.05 – 1.56)	**0.014**	1.20 (0.98 – 1.47)	0.073
Site of Primary				
**Oropharynx**	Reference		Reference	
**Oral Cavity**	1.64 (1.42 – 1.90)	**< 0.001**	1.18 (0.94 – 1.47)	0.146
**Larynx**	1.25 (1.08 – 1.44)	**0.002**	0.86 (0.71 – 1.05)	0.149
**Hypopharynx**	1.09 (0.85 – 1.41)	0.483	0.87 (0.65 – 1.16)	0.331
**H&N Unknown Primary**	0.53 (0.32 – 0.89)	**0.016**	0.40 (0.24 – 0.69)	**0.001**
Advanced Criteria				
**Distant Metastatic**	Reference		Reference	
**Locoregional Recurrence**	1.19 (1.06 – 1.34)	**0.003**	1.09 (0.97 – 1.24)	0.125
HPV Association				
**HPV associated**	Reference		Reference	
**Non-HPV associated**	1.57 (1.38 – 1.79)	**< 0.001**	1.48 (1.21 – 1.80)	**< 0.001**
**Unknown**	1.67 (1.30 – 2.15)	**< 0.001**	1.51 (1.16 – 1.97)	**0.002**
Smoking Status				
**Smoker**	Reference		Reference rR	
**Non-Smoker**	0.88 (0.76 – 1.01)	**0.061**	0.93 (0.81 – 1.08)	0.360
Primary Surgery				
**Yes**	Reference		Reference	
**No**	0.84 (0.75 – 0.95)	**0.005**	0.94 (0.82 – 1.08)	0.365
Primary Radiation				
**Yes**	Reference		Reference	
**No**	1.18 (1.03 – 1.34)	**0.014**	1.08 (0.94 – 1.24)	0.256
Treatment				
**anti-PD1 Monotherapy**	Reference			
**anti-PD1 + Chemo**	0.97 (0.85 – 1.10)	0.611		
CPS				
**< 1**	Reference		Reference	
**1-9**	1.06 (0.86 – 1.31)	0.601	0.92 (0.74 – 1.15)	0.474
**10-19**	1.11 (0.84 – 1.47)	0.474	1.03 (0.78 – 1.37)	0.814
**≥20**	1.00 (0.83 – 1.22)	0.965	0.90 (0.74 – 1.09)	0.266
**NA**	1.15 (1.00 – 1.33)	**0.053**	1.09 (0.94 – 1.26)	0.250

Bolded values represent statistically significant p-values at a threshold of p < 0.05.

## Discussion

In this study, we examine real world practice patterns and outcomes in the treatment of recurrent or metastatic HNSCC in the immunotherapy era. Overall survival in patients with non-HPV associated tumors were comparable to the findings in KN048 when looking at all CPS groups. The median overall survival of each of the treatment arms was generally similar between our study and KN048 (12.6 vs 10.7 mos for Cetuximab plus chemotherapy, 14.6 vs 11.5 mos for anti-PD1 alone, and 14.3 vs 13.0 mos for anti-PD1 plus chemotherapy respectively), although this is not directly comparable due to key differences in the patient populations such as the real world and retrospective nature of our data along with the higher proportion of HPV-associated disease in our study (38.8% in our study versus about 21% in KN048) (1, 2). However, anti-PD1 plus chemotherapy did not have significantly improved survival over cetuximab + chemotherapy in the overall population as it was demonstrated in KN048, although it strongly trended towards this. This is likely due to the non-randomized and retrospective nature of our analysis, including a highly probable selection bias where clinical factors associated with worse prognosis were associated with the decision to add chemotherapy to immunotherapy. Additionally, treatment sequencing likely plays a role in this discrepancy as 52.3% of patients in our study treated front-line with Cetuximab + chemotherapy received subsequent immunotherapy containing treatment regimens whereas about only 25% of similar patients in KN048 received immunotherapy containing regimens (2). This higher rate of subsequent immunotherapy treatment may have improved survival of this treatment group in our study.

HPV-associated oropharyngeal squamous cell carcinoma has emerged as unique subtype of HNSCC with improved survival (7). Interestingly in our study, patients with HPV-associated tumors did not demonstrate differences in survival between first-line anti-PD1 monotherapy, combination anti-PD1 plus chemotherapy, and cetuximab plus chemotherapy. This finding is consistent with the results of Checkmate 651, where subset analyses demonstrated no significant difference in survival between ipilimumab + nivolumab versus the EXTREME regimen in patients with p16+ oropharynx cancer, with a shorter median survival of 19.8 months with ipilimumab + nivolumab versus 23.8 months with the EXTREME regimen (8). On the other hand, while subset analyses of KN048 of patients with p16 positive disease did not demonstrate improvement in survival with pembrolizumab monotherapy (HR 0.80, 95% CI 0.53 – 1.20), they did suggest improved survival with combination anti-PD1 plus chemotherapy (HR 0.56, 95% CI 0.36 – 0.87) which differs from the results of our study (1). It is unclear whether this is a result of the retrospective nature of our study, clinical factors that influenced treatment choice, the relatively small number of patients with HPV associated disease in KN048, or the longer remaining life expectancy of p16+ disease in which to receive additional lines of therapy.

In our subset analyses, patients with non-HPV associated disease and tumor CPS 1-9 significantly benefitted from the addition of chemotherapy to anti-PD1 while those with CPS < 1 or ≥10 did not. It is possible that for patients with recurrent or metastatic non-HPV associated head and neck cancer and CPS 1-9, the addition of chemotherapy to anti-PD1 therapy may compensate for reduced sensitivity to immunotherapy either through additive or synergistic effects, although further prospective investigation of this cutoff is warranted. Although a different disease context, a CPS cutoff of ≥10 is also a clinically meaningful predictor of response to pembrolizumab therapy in the setting of metastatic HER2 negative gastroesophageal squamous cell carcinoma and adenocarcinoma as seen in KEYNOTE-859; this may highlight CPS ≥10 as a potentially meaningful across different malignancies and nominates this group for further study within head and neck cancer (13). Given the significantly increased rates of adverse events with the addition of chemotherapy to anti-PD1 treatment, the development of a clear CPS cutoff for a subset of patients may allow for certainty with treatment regimen selection while weighing risks and benefits.

On the other hand, patients with HPV-associated oropharyngeal cancer did not have differences in survival between anti-PD1 monotherapy and combination anti-PD1 plus chemotherapy in any CPS subset. Some studies such as a *post hoc* analysis of Checkmate-141 have suggested improved efficacy of anti-PD1 therapy in HPV associated disease, which might explain this finding, while other studies have seen no difference by HPV status as seen in KEYNOTE-012 and KEYNOTE-055 (9–12). Our results may support further investigation of treatment de-escalation for HPV-positive disease through the use of the less toxic anti-PD1 monotherapy regimen given the equivalent outcomes of all 3 treatment regimens in our study.

For patients with recurrent or metastatic HNSCC, primary tumors of the oral cavity have been previously associated with worse outcomes particularly compared to primary tumors of the oropharynx (14). However, this is the first study to demonstrate improved survival with combination anti-PD1 plus chemotherapy for patients with primary tumors of the oral cavity compared to anti-PD1 monotherapy. As seen in our study, primary tumor site is not a factor associated with the treatment regimen chosen for recurrent or metastatic disease. Perhaps the differential response to anti-PD1 monotherapy versus combination therapy for oral cavity primaries, but not for primary tumors of other sites, suggests differing underlying biology that should impact the choice of front-line treatment. Given that most oral cavity primaries are non-HPV associated and non-HPV associated tumors of the oropharynx did not demonstrate a difference in survival by treatment regimen in our study, perhaps this biological difference extends beyond HPV status.

Our study has several noteworthy findings pertaining to the impact of demographics and social determinants of health on treatment patterns and survival outcomes. With regards to treatment patterns, patient demographics such as sex, race, and socioeconomic status were associated with the choice between anti-PD1 monotherapy and combination anti-PD1 plus chemotherapy. Male patients and patients of higher socioeconomic status were more likely to receive anti-PD1 plus chemotherapy, while patients with race listed as “other” were significantly less likely to receive chemotherapy compared to white patients. With regards to overall survival, Hispanic patients had significantly improved survival compared to non-Hispanic patients in our study which is consistent with prior studies (15).

Key limitations of this study include its retrospective nature. While the accuracy of the treatment line variable in this dataset has been internally validated by Flatiron, it is possible that first-line treatment may have been miscoded in this dataset if first-line treatment took place outside of an affiliated practice. The variables of locoregionally recurrent disease and distant metastatic disease were coded as mutually exclusive in the dataset, so there are likely patients with both locoregionally recurrent and distant metastatic disease that could not be further evaluated. While our multivariable models accounted for the listed covariates, it is possible that other confounders may impact survival analyses and analysis of factors associated with treatment regimen. There is a notable population of patients with missing CPS information which may have impacted and limited the power of subset analyses.

## Conclusion

Our retrospective real-world study recapitulates many findings of KN048. Certain subsets of patients, including patients with oral cavity primaries and those with both non-HPV associated disease and CPS 1-9, may benefit from addition of chemotherapy to anti-PD1. HPV associated disease did not have differences in outcomes by treatment regimen, potentially supporting treatment de-escalation for this cohort. These findings warrant further prospective studies.

## Data Availability

The data that support the findings of this study were originated by and are the property of Flatiron Health, Inc. Requests for data sharing by license or by permission for the specific purpose of replicating results in this manuscript can be submitted to https://PublicationsDataAccess@flatiron.com.

## Supplementary material

Supplementary Figure 1 CONSORT diagram. H&N, head and neck; CONSORT, Consolidated Standards of Reporting Trials.

Supplementary Figure 2 Survival by treatment regimen for patients with CPS1-19 in the **(A)** overall population, **(B)** non-HPV associated population, and **(C)** HPV associated population. P values are based on log rank test. HPV, human papilloma virus; CPS, combined positive score; Chemo, chemotherapy.

Supplementary Figure 3 Survival by CPS within each treatment regimen and by HPV association. Overall survival for patients by CPS <1, 1-9, and ≥10 for patients treated with **(A)** anti-PD1 monotherapy and **(B)** anti-PD1 plus chemotherapy. Overall survival by treatment regimen for patients with **(C, D)** HPV associated tumors and **(E, F)** non-HPV associated tumors. P values are based on log rank test. aPD1, anti-PD1; HPV, human papilloma virus; CPS, combined positive score; Chemo, chemotherapy.

Supplementary Figure 4 Primary tumors of the oral cavity have worse outcomes in the recurrent or metastatic setting. Overall survival for patients with oral cavity primary tumors versus patients with **(A)** oropharyngeal primary tumors (HPV associated and non-HPV associated), **(B)** laryngeal primary tumors, **(C)** hypopharyngeal primary tumors, and **(D)** head and neck cancer with unknown primary. P values are based on log rank test. HPV. human papilloma virus; H&N. Head and Neck.
