# Supplementary figures and images for: Real world treatment patterns for recurrent and metastatic head and neck cancer in the post-KEYNOTE 048 era
Source: Front Oncol. 2025 May 2;15:1577509. doi: 10.3389/fonc.2025.1577509 (PMC12099209; doi:10.3389/fonc.2025.1577509)

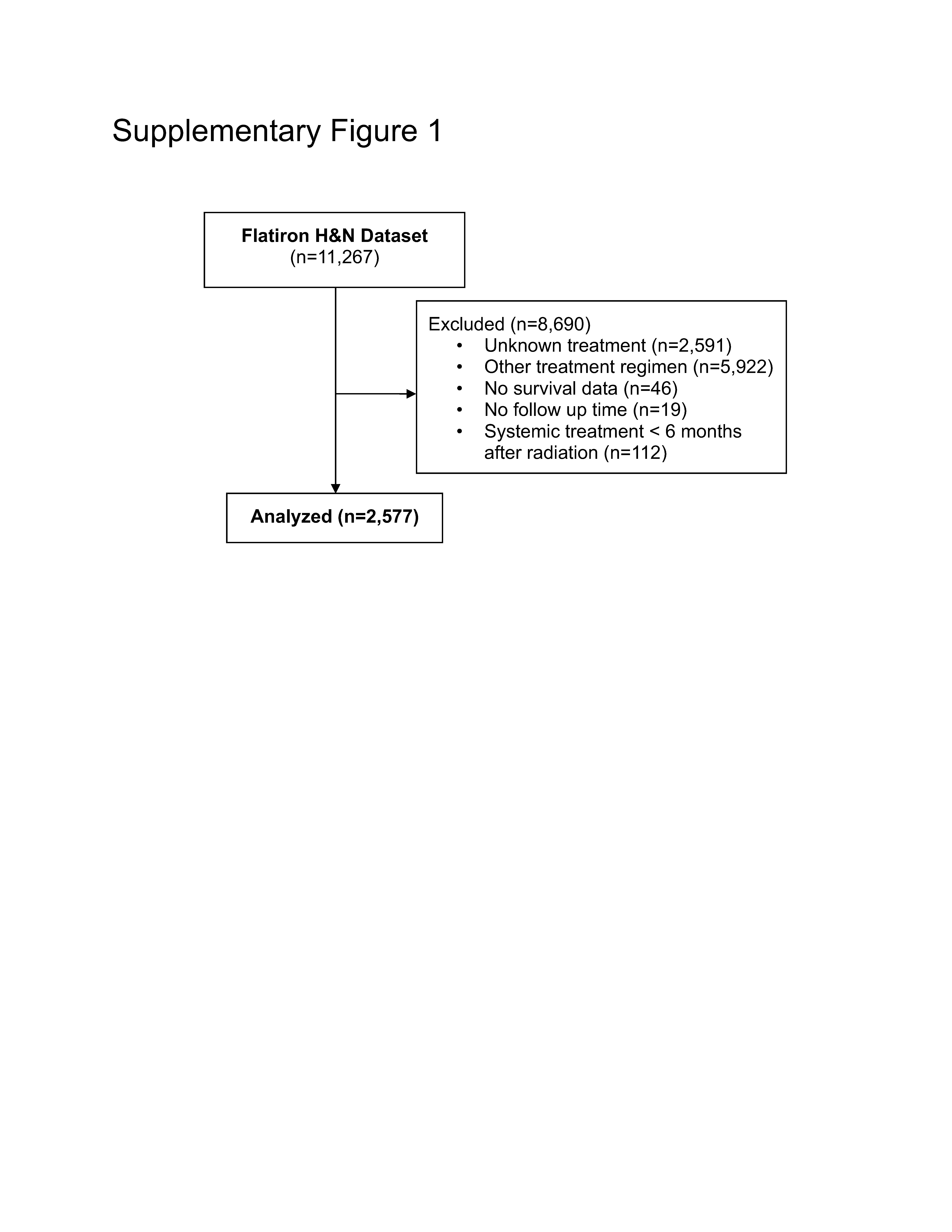

Supplement: Supplementary Figure 1 — CONSORT diagram. H&N, head and neck; CONSORT, Consolidated Standards of Reporting Trials. [file Image1.jpeg]

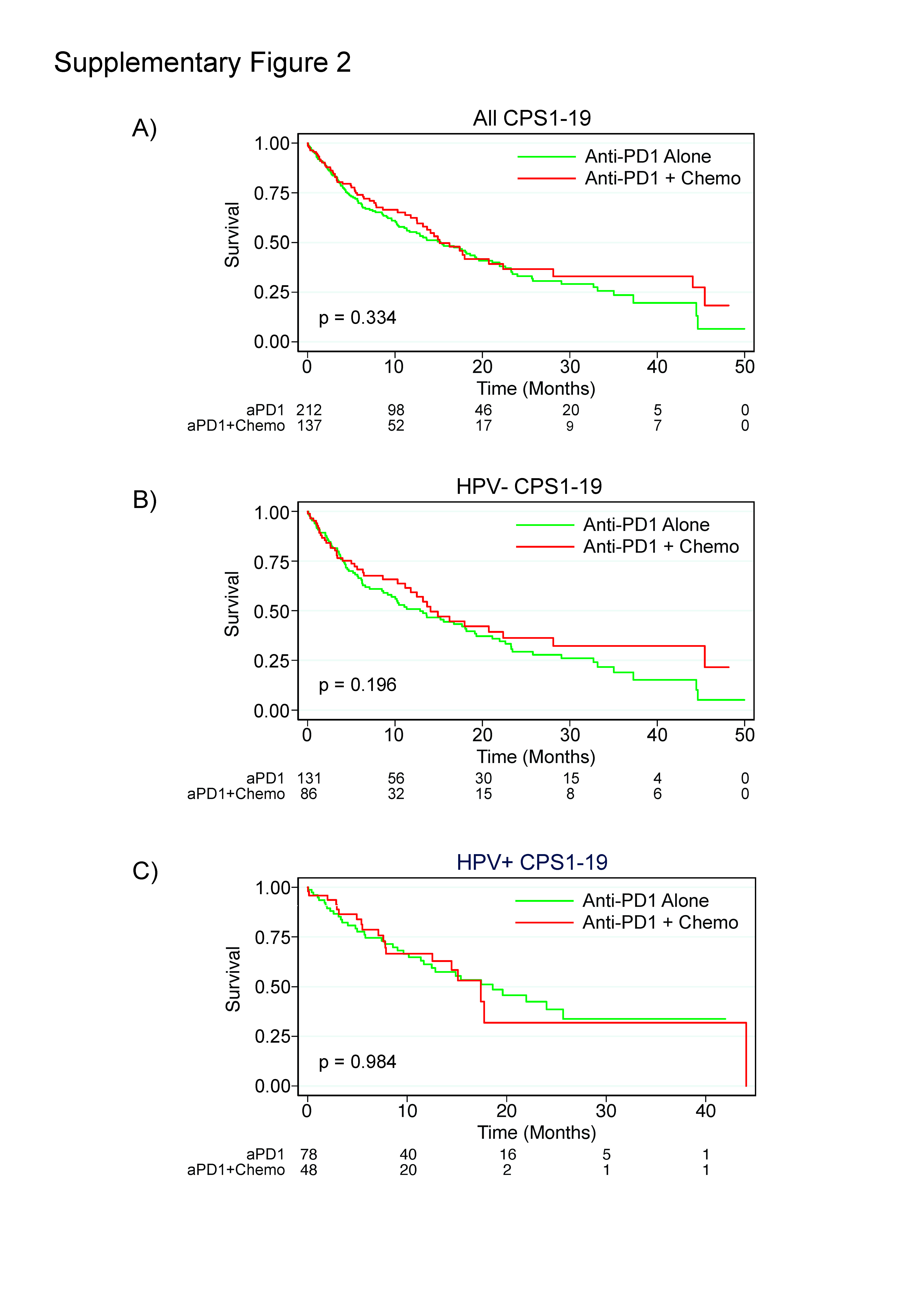

Supplement: Supplementary Figure 2 — Survival by treatment regimen for patients with CPS1-19 in the (A) overall population, (B) non-HPV associated population, and (C) HPV associated population. P values are based on log rank test. HPV, human papilloma virus; CPS, combined positive score; Chemo, chemotherapy. [file Image2.jpeg]

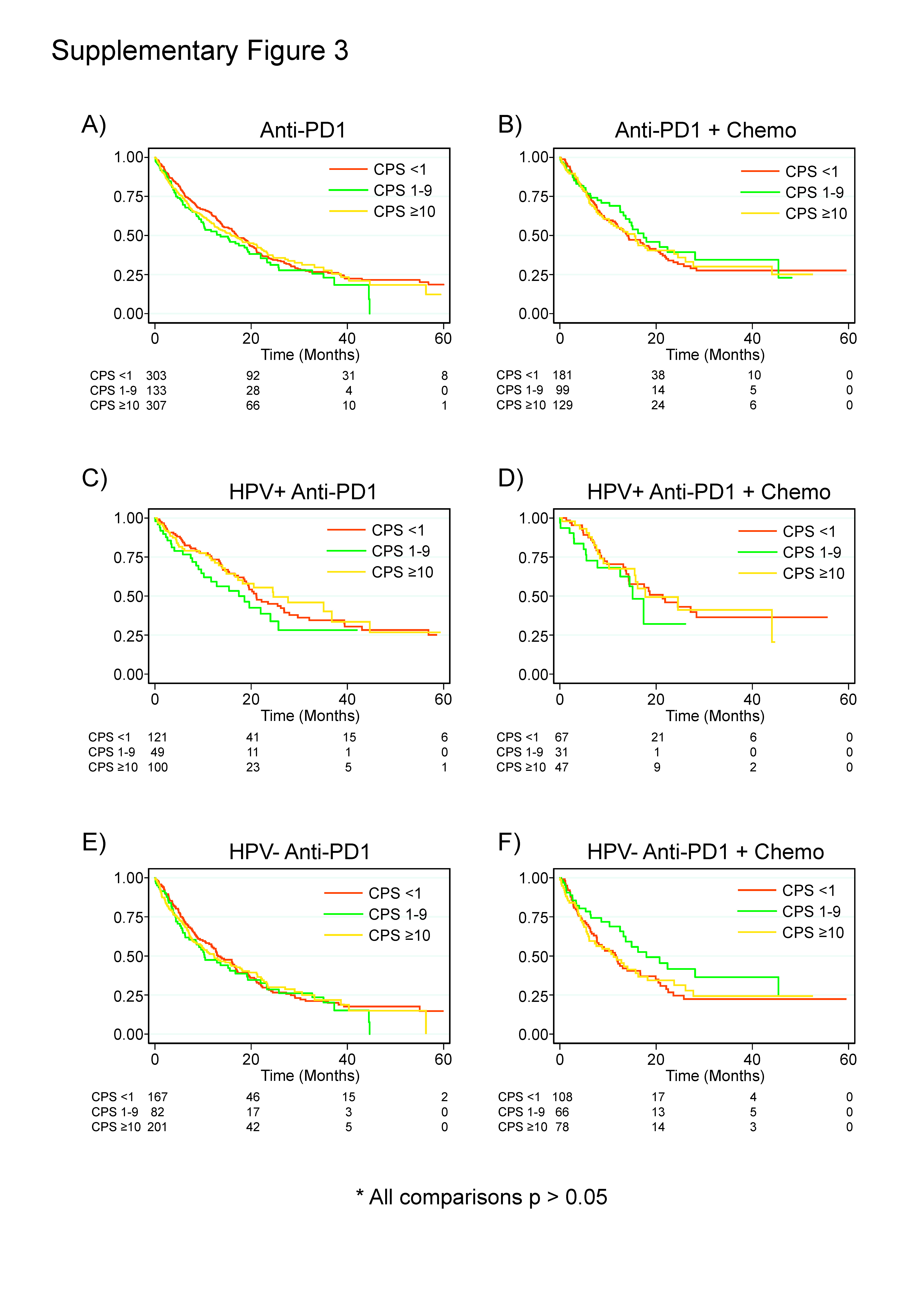

Supplement: Supplementary Figure 3 — Survival by CPS within each treatment regimen and by HPV association. Overall survival for patients by CPS <1, 1-9, and ≥10 for patients treated with (A) anti-PD1 monotherapy and (B) anti-PD1 plus chemotherapy. Overall survival by treatment regimen for patients with (C, D) HPV associated tumors and (E, F) non-HPV associated tumors. P values are based on log rank test. aPD1, anti-PD1; HPV, human papilloma virus; CPS, combined positive score; Chemo, chemotherapy. [file Image3.jpeg]

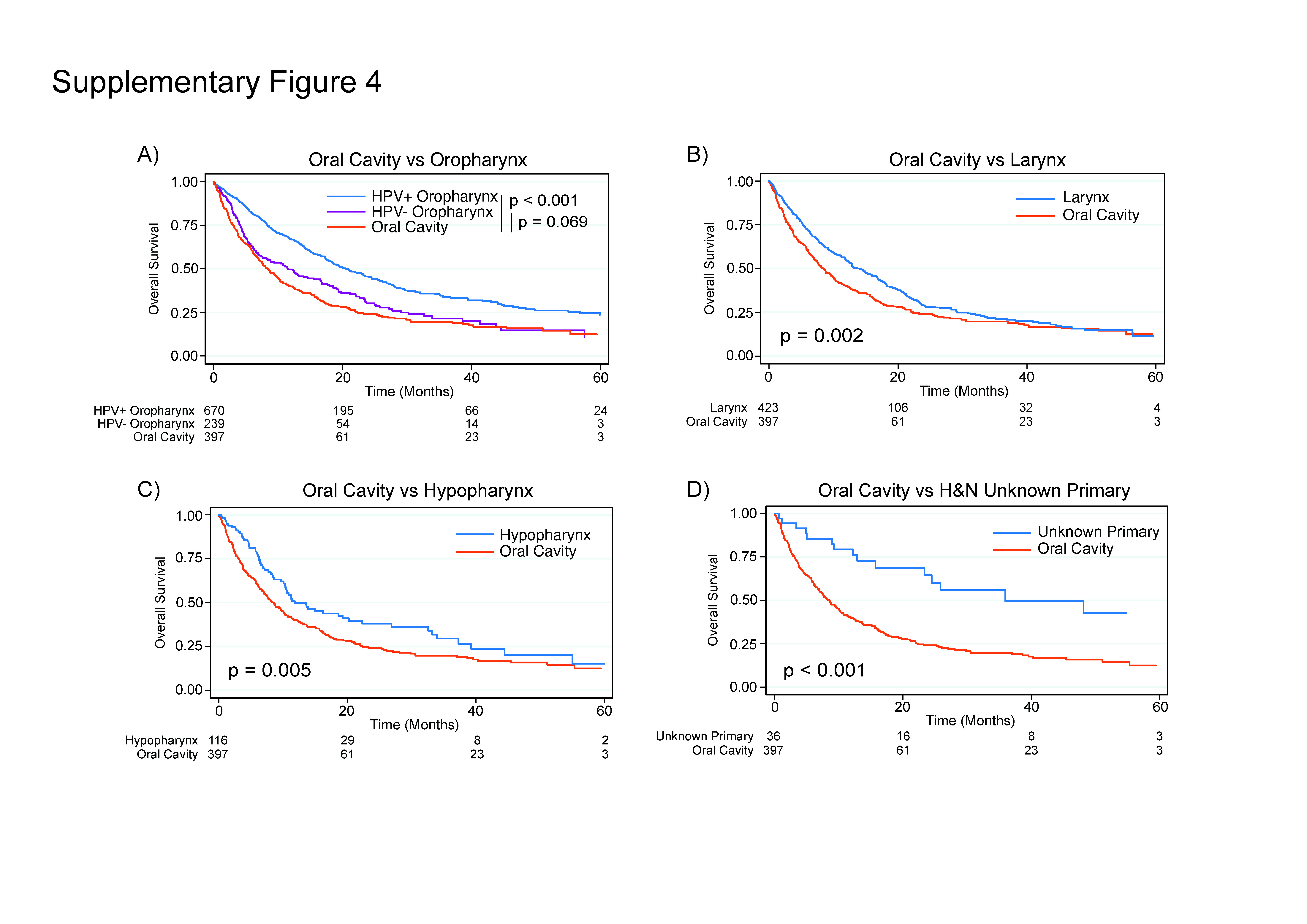

Supplement: Supplementary Figure 4 — Primary tumors of the oral cavity have worse outcomes in the recurrent or metastatic setting. Overall survival for patients with oral cavity primary tumors versus patients with (A) oropharyngeal primary tumors (HPV associated and non-HPV associated), (B) laryngeal primary tumors, (C) hypopharyngeal primary tumors, and (D) head and neck cancer with unknown primary. P values are based on log rank test. HPV. human papilloma virus; H&N. Head and Neck. [file Image4.jpeg]
