# Supplementary material for: Real world treatment patterns for recurrent and metastatic head and neck cancer in the post-KEYNOTE 048 era
Source: Front Oncol. 2025 May 2;15:1577509. doi: 10.3389/fonc.2025.1577509 (PMC12099209; doi:10.3389/fonc.2025.1577509)
Supplement: Supplementary file 5 [file Table1.docx]

| Supp Table 1: IO Treatment Regimens | |
| --- | --- |
| Treatment Name | **Patients** |
| Pembrolizumab | 1,051 |
| Nivolumab | 359 |
| Pembro + Carboplatin/5-FU | 324 |
| Pembro + Carboplatin/Paclitaxel | 157 |
| Pembro + Cisplatin/5-FU | 64 |
| Pembro + Carboplatin/Docetaxel | 17 |
| Pembro + Cisplatin/Paclitaxel | 5 |
| Pembro + Carboplatin/Docetaxel/5-FU | 4 |
| Pembro + Cisplatin/Docetaxel/5-FU | 3 |
| Pembro + Carboplatin/Docetaxel/Paclitaxel | 1 |
| Pembro + Oxaliplatin/Docetaxel/5-FU | 1 |
| Nivo + Carboplatin/Docetaxel | 1 |

| Supp Table 2: Cetuximab Treatment Regimens | |
| --- | --- |
| Treatment Name | **Patients** |
| Cetuximab + Carboplatin/Paclitaxel | 234 |
| Cetuximab + Carboplatin/5-FU | 209 |
| Cetuximab + Cisplatin/5-FU | 77 |
| Cetuximab + Carboplatin/Docetaxel | 27 |
| Cetuximab + Cisplatin/Paclitaxel | 14 |
| Cetuximab + Cisplatin/Docetaxel | 9 |
| Cetuximab + Cisplatin/Docetaxel/5-FU | 9 |
| Cetuximab + Carboplatin/Docetaxel/5-FU | 5 |
| Cetuximab + Cisplatin/Paclitaxel/5-FU | 2 |
| Cetuximab + Carboplatin/Paclitaxel/5-FU | 2 |
| Cetuximab + Carboplatin/Docetaxel/Paclitaxel | 1 |
| Nivo + Carboplatin/Docetaxel | 1 |
