# Supplementary material for: Real world treatment patterns for recurrent and metastatic head and neck cancer in the post-KEYNOTE 048 era
Source: Front Oncol. 2025 May 2;15:1577509. doi: 10.3389/fonc.2025.1577509 (PMC12099209; doi:10.3389/fonc.2025.1577509)
Supplement: Supplementary file 6 [file Table2.docx]

| Supplementary Table 3: Second Line Treatment Regimen Summary | | | | |
| --- | --- | --- | --- | --- |
| **Treatment Regimen** | **Total Patients** | **Anti-PD1** | **Anti-PD1 + chemo** | **Cetux + chemo** |
| Anti-PD1 monotherapy | 240 | 62 | 10 | 168 |
| Anti-PD1 + platinum + 5FU/taxane | 93 | 66 | 18 | 9 |
| Cetuximab + platinum + 5FU/taxane | 140 | 69 | 43 | 28 |
| Anti-PD1 + Cetuximab | 16 | 7 | 5 | 4 |
| Anti-PD1 + Cetuximab + chemo | 11 | 5 | 3 | 3 |
| Cetuximab alone | 116 | 87 | 24 | 5 |
| Anti-PD1 + other chemo | 31 | 24 | 3 | 4 |
| Cetuximab + other chemo | 51 | 25 | 14 | 12 |
| Other chemo | 325 | 175 | 45 | 105 |
| Anti-PD1 + Anti-CTLA4 | 3 | 3 | 0 | 0 |
| Anti-PD1 + Anti-CTLA4 + chemo | 1 | 1 | 0 | 0 |
| Anti-PD1 + other | 17 | 13 | 1 | 3 |
| Cetuximab + other | 12 | 3 | 7 | 2 |
| Other | 72 | 32 | 18 | 22 |
| Total | 1128 | 572 | 191 | 365 |
