# Supplementary material for: Real world treatment patterns for recurrent and metastatic head and neck cancer in the post-KEYNOTE 048 era
Source: Front Oncol. 2025 May 2;15:1577509. doi: 10.3389/fonc.2025.1577509 (PMC12099209; doi:10.3389/fonc.2025.1577509)
Supplement: Supplementary file 7 [file Table3.docx]

| Supplementary Figure 4: Reported Second Line Treatment Regimens | |
| --- | --- |
| Treatment | **Count** |
| Pembrolizumab | 150 |
| Cetuximab | 116 |
| Carboplatin,Paclitaxel | 110 |
| Nivolumab | 89 |
| Carboplatin,Cetuximab,Paclitaxel | 67 |
| Clinical Study Drug | 58 |
| Docetaxel | 48 |
| Carboplatin,Paclitaxel,Pembrolizumab | 42 |
| Carboplatin,Cetuximab,Fluorouracil | 37 |
| Paclitaxel | 35 |
| Carboplatin,Fluorouracil,Pembrolizumab | 24 |
| Carboplatin,Docetaxel | 17 |
| Carboplatin,Cetuximab,Docetaxel | 16 |
| Methotrexate | 16 |
| Cisplatin | 14 |
| Cetuximab,Paclitaxel | 13 |
| Carboplatin,Cetuximab | 13 |
| Capecitabine | 13 |
| Cetuximab,Clinical Study Drug | 11 |
| Carboplatin,Pembrolizumab | 11 |
| Cetuximab,Cisplatin | 9 |
| Cetuximab,Pembrolizumab | 9 |
| Carboplatin | 8 |
| Gemcitabine | 8 |
| Cisplatin,Fluorouracil,Pembrolizumab | 7 |
| Cetuximab,Cisplatin,Fluorouracil | 7 |
| Cisplatin,Docetaxel | 6 |
| Cetuximab,Docetaxel | 6 |
| Cetuximab,Nivolumab | 5 |
| Carboplatin,Docetaxel,Pembrolizumab | 5 |
| Clinical Study Drug,Pembrolizumab | 5 |
| Cisplatin,Docetaxel,Fluorouracil | 5 |
| Paclitaxel Protein-Bound | 5 |
| Cisplatin,Fluorouracil | 4 |
| Carboplatin,Fluorouracil | 4 |
| Cetuximab,Methotrexate | 4 |
| Carboplatin,Paclitaxel Protein-Bound | 3 |
| Cisplatin,Paclitaxel | 3 |
| Paclitaxel,Pembrolizumab | 3 |
| Carboplatin,Cetuximab,Paclitaxel,Pembrolizumab | 3 |
| Carboplatin,Paclitaxel Protein-Bound,Pembrolizumab | 3 |
| Carboplatin,Cetuximab,Paclitaxel Protein-Bound | 3 |
| Carboplatin,Nivolumab,Paclitaxel | 3 |
| Ipilimumab,Nivolumab | 3 |
| Vinorelbine | 3 |
| Cetuximab,Gemcitabine | 2 |
| Gemcitabine,Methotrexate | 2 |
| Fluorouracil,Pembrolizumab | 2 |
| Carboplatin,Cetuximab,Docetaxel,Paclitaxel | 2 |
| Clinical Study Drug,Nivolumab | 2 |
| Carboplatin,Docetaxel,Fluorouracil | 2 |
| Clinical Study Drug,Docetaxel | 2 |
| Fluorouracil | 2 |
| Cetuximab,Cisplatin,Docetaxel | 2 |
| Carboplatin,Nivolumab | 2 |
| Afatinib | 2 |
| Cisplatin,Paclitaxel Protein-Bound | 2 |
| Carboplatin,Fluorouracil,Paclitaxel,Pembrolizumab | 2 |
| Cetuximab,Nivolumab,Pembrolizumab | 2 |
| Carboplatin,Cetuximab,Fluorouracil,Pembrolizumab | 2 |
| Capecitabine,Carboplatin,Pembrolizumab | 2 |
| Enzalutamide,Leuprolide | 1 |
| Fulvestrant,Pembrolizumab | 1 |
| Ifosfamide,Mesna,Paclitaxel | 1 |
| Methotrexate,Pembrolizumab | 1 |
| Cetuximab,Oxaliplatin | 1 |
| Fluorouracil,Oxaliplatin,Pembrolizumab | 1 |
| Cabozantinib | 1 |
| Axitinib,Pembrolizumab | 1 |
| Carboplatin,Etoposide | 1 |
| Cabozantinib,Clinical Study Drug | 1 |
| Capecitabine,Carboplatin,Paclitaxel,Pembrolizumab | 1 |
| Pembrolizumab,Rituximab | 1 |
| Cetuximab,Fluorouracil,Oxaliplatin | 1 |
| Docetaxel,Gemcitabine | 1 |
| Cetuximab,Nivolumab,Paclitaxel | 1 |
| Cisplatin,Gemcitabine | 1 |
| Idelalisib | 1 |
| Afatinib,Cetuximab,Clinical Study Drug | 1 |
| Cyclophosphamide,Doxorubicin,Rituximab-Pvvr,Rituximab/Hyaluronidase,Vincristine | 1 |
| Gemcitabine,Paclitaxel | 1 |
| Gemcitabine,Pembrolizumab | 1 |
| Bortezomib,Cyclophosphamide,Pembrolizumab | 1 |
| Cisplatin,Fluorouracil,Nivolumab,Pembrolizumab | 1 |
| Docetaxel,Pembrolizumab | 1 |
| Carboplatin,Gemcitabine | 1 |
| Cetuximab,Gemcitabine,Pembrolizumab | 1 |
| Abiraterone,Pembrolizumab | 1 |
| Capecitabine,Cisplatin,Pembrolizumab | 1 |
| Cetuximab,Fluorouracil | 1 |
| Capecitabine,Cetuximab,Nivolumab | 1 |
| Gemcitabine,Paclitaxel Protein-Bound | 1 |
| Carboplatin,Gemcitabine,Nivolumab | 1 |
| Letrozole | 1 |
| Carboplatin,Cetuximab,Fluorouracil,Paclitaxel Protein-Bound | 1 |
| Amifostine,Carboplatin,Cetuximab,Paclitaxel | 1 |
| Fluorouracil,Leucovorin,Levoleucovorin,Oxaliplatin | 1 |
| Cetuximab,Gemcitabine,Methotrexate | 1 |
| Erlotinib | 1 |
| Bendamustine,Rituximab-Pvvr | 1 |
| Cisplatin,Paclitaxel,Pembrolizumab | 1 |
| Capecitabine,Cetuximab,Pembrolizumab | 1 |
| Cetuximab,Paclitaxel,Pembrolizumab | 1 |
| Nivolumab,Pembrolizumab | 1 |
| Hydroxyurea,Nivolumab | 1 |
| Carboplatin,Clinical Study Drug,Paclitaxel | 1 |
| Carboplatin,Docetaxel,Ipilimumab,Nivolumab | 1 |
| Carboplatin,Fluorouracil,Leucovorin,Pembrolizumab | 1 |
| Daratumumab/Hyaluronidase-Fihj,Nivolumab | 1 |
| Cisplatin,Docetaxel,Fluorouracil,Leucovorin | 1 |
| Cisplatin,Pembrolizumab | 1 |
| Anastrozole,Nivolumab | 1 |
| Fluorouracil,Levoleucovorin | 1 |
| Fluorouracil,Leucovorin,Pembrolizumab | 1 |
| Capecitabine,Carboplatin,Cetuximab | 1 |
| Carboplatin,Fluorouracil,Leucovorin | 1 |
| Lenvatinib | 1 |
| Azacitidine,Nivolumab | 1 |
| Carboplatin,Necitumumab,Paclitaxel,Pembrolizumab | 1 |
| Cisplatin,Nivolumab | 1 |
| Palbociclib | 1 |
| Fluorouracil,Leucovorin | 1 |
| Panitumumab | 1 |
| Cetuximab,Paclitaxel Protein-Bound,Panitumumab,Pembrolizumab | 1 |
| Olaparib | 1 |
| Cetuximab,Cisplatin,Docetaxel,Fluorouracil | 1 |
| Cisplatin,Docetaxel,Fluorouracil,Pembrolizumab | 1 |
| Carboplatin,Oxaliplatin,Paclitaxel,Pembrolizumab | 1 |
| Erdafitinib,Nivolumab | 1 |
| Carboplatin,Cetuximab,Fluorouracil,Paclitaxel | 1 |
| Carboplatin,Clinical Study Drug,Paclitaxel,Pembrolizumab | 1 |
| Carboplatin,Pembrolizumab,Pemetrexed | 1 |
| Cetuximab,Cisplatin,Paclitaxel | 1 |
